# Development of In Situ Gelling Meloxicam-Human Serum Albumin Nanoparticle Formulation for Nose-to-Brain Application

**DOI:** 10.3390/pharmaceutics13050646

**Published:** 2021-05-01

**Authors:** Gábor Katona, Bence Sipos, Mária Budai-Szűcs, György Tibor Balogh, Szilvia Veszelka, Ilona Gróf, Mária A. Deli, Balázs Volk, Piroska Szabó-Révész, Ildikó Csóka

**Affiliations:** 1 Institute of Pharmaceutical Technology and Regulatory Affairs, Faculty of Pharmacy, University of Szeged, Eötvös Str. 6, H-6720 Szeged, Hungary; sipos.bence@szte.hu (B.S.); budai-szucs.maria@szte.hu (M.B.-S.); ReveszPiroska@szte.hu (P.S.-R.); csoka.ildiko@szte.hu (I.C.); 2 Department of Pharmacodynamics and Biopharmacy, Faculty of Pharmacy, University of Szeged, Eötvös Str. 6, H-6720 Szeged, Hungary; balogh.gyorgy.tibor@szte.hu; 3 Department of Chemical and Environmental Process Engineering, Budapest University of Technology and Economics, Műegyetem Quay 3, H-1111 Budapest, Hungary; 4 Biological Research Centre, Institute of Biophysics, Temesvári Blvd. 62, H-6726 Szeged, Hungary; veszelka.szilvia@brc.hu (S.V.); grof.ilona@brc.hu (I.G.); deli.maria@brc.hu (M.A.D.); 5 Egis Pharmaceuticals Plc., Keresztúri Str. 30–38, H-1106 Budapest, Hungary; volk.balazs@egis.hu

**Keywords:** quality by design, rapid equilibrium dialysis, muco-adhesion, brain PAMPA, RPMI 2650 nasal epithelial cell

## Abstract

The aim of this study was to develop an intranasal in situ thermo-gelling meloxicam-human serum albumin (MEL-HSA) nanoparticulate formulation applying poloxamer 407 (P407), which can be administered in liquid state into the nostril, and to increase the resistance of the formulation against mucociliary clearance by sol-gel transition on the nasal mucosa, as well as to improve drug absorption. Nanoparticle characterization showed that formulations containing 12–15% *w*/*w* P407 met the requirements of intranasal administration. The Z-average (in the range of 180–304 nm), the narrow polydispersity index (PdI, from 0.193 to 0.328), the zeta potential (between −9.4 and −7.0 mV) and the hypotonic osmolality (200–278 mOsmol/L) of MEL-HSA nanoparticles predict enhanced drug absorption through the nasal mucosa. Based on the rheological, muco-adhesion, drug release and permeability studies, the 14% *w*/*w* P407 containing formulation (MEL-HSA-P14%) was considered as the optimized formulation, which allows enhanced permeability of MEL through blood–brain barrier-specific lipid fraction. Cell line studies showed no cell damage after 1-h treatment with MEL-HSA-P14% on RPMI 2650 human endothelial cells’ moreover, enhanced permeation (four-fold) of MEL from MEL-HSA-P14% was observed in comparison to pure MEL. Overall, MEL-HSA-P14% can be promising for overcoming the challenges of nasal drug delivery.

## 1. Introduction

Albumin is a versatile, biodegradable drug carrier for numerous therapeutic agents that have poor water solubility, unsatisfying pharmacokinetics with low circulation half-life, inefficient targetability and even instability in vivo. Strategies for applying albumin for drug delivery can be classified broadly into exogenous and in situ binding formulations that utilize covalent attachment, non-covalent association, or encapsulation of the drug in the form of albumin-based nanoparticles [1].

Neurodegenerative diseases are associated with neuroinflammation. The combination of albumin with non-steroid anti-inflammatory drugs (NSAID) can be promising in therapy, which depends on passing the blood–brain barrier (BBB). NSAIDs can have a protective effect in neurodegenerative diseases through different mechanisms. They can depolarize the mitochondria, therefore inhibiting calcium ion uptake, due to the ionizable carboxylic group [2,3]. Moreover, inhibition of cyclooxygenase (COX) enzymes can suppress glia activity and reduce amyloidosis [4]. COX-2 inhibitor NSAIDs such as meloxicam (MEL) can be advantageous in the treatment of neurodegenerative disorders as they have improved anti-amnesic activity through inhibiting lipid peroxidation and acetylcholinesterase activity in the brain [2] supplemented by additional antioxidant effect [3], but the therapeutic application is limited due to the poor BBB transport [5]. To overcome this obstacle, choosing the appropriate carrier system and route of administration has a prominent role [6].

Nasal administration can be a suitable means of transport route for that purpose; moreover, it has been reported that the initial formation of Alzheimer’s disease begins in the entorhinal cortex, a region innervated by the olfactory nerves, then progresses according to the corresponding pattern [7]. Due to the high surface area and rich vascularization, drugs or drug-delivery systems can be easily absorbed from the nasal cavity; moreover, first-pass metabolism is negligible through this administration route, which can be advantageous in terms of preserving pharmacological activity [8,9,10]. Nano drug delivery systems (nanoDDSs) are able to transport drugs as cargo, bypassing the BBB through the trigeminal and olfactory nerves directly into the brain [11]. Moreover, they support intranasal NSAID administration, due to their poor water solubility at nasal pH (5.3–5.6) and low residence time (10–15 min) due to mucociliary clearance [12]. In our previous study was demonstrated that MEL-human serum albumin (HSA) nanoparticles could be successfully applied for nose-to-brain delivery, with improved in vivo brain targeting efficacy [13].

As mucociliary clearance is a limiting factor in nose-to-brain delivery, the application of viscosity enhancers or mucoadhesive polymers can be advantageous by increasing the residence time on the nasal mucosa, which supports drug absorption [14,15,16]. To satisfy these requirements formulation of in situ thermo-gelling systems can be efficacious. Poloxamer 407 (P407), a triblock copolymer consisting of a hydrophobic residue of poly-oxy-propylene (POP) between the two hydrophilic units of poly-oxyethylene (POE), can be applied for development of in situ thermo-reversible gelling systems, through temperature-controlled micelle forming [17,18,19]. Thermo-gelling occurs due to hydrophobic interactions between the P407 copolymer chains [20]. By optimization of P407 concentration in the formulation, a sol–gel transition can be reached at the temperature of the nasal cavity, while it remains in a liquid state below that temperature during storage and administration.

As continuous improvement is part of industrial manufacturing and research processes, the quality management of nanocarriers having higher potential, in the case of beneficial therapeutic applications and effects, should be of paramount importance. Quality by Design (QbD) offers a proper methodology based on knowledge and risk assessment, ensuring the quality, safety and efficacy of the desirable nanocarrier [21]. In the case of nose-to-brain applicable nanocarriers, many physiological and pharmaceutical aspects must be taken into account, which are adapted to the versatile biological and chemical aspects of this route. As part of the QbD assessment, a comparison study was performed between MEL-HSA and MEL-HSA-P407 formulations to evaluate the change of risk severity during a continuous development process [22].

Our aim was to optimize an in situ thermo-gelling MEL-HSA-P407 formulation, which can be administered in liquid state into the nostril and to increase resistance of formulation against mucociliarly clearance by sol–gel transition on the nasal mucosa, as well as to improve drug absorption.

## 2. Materials and Methods

### 2.1. Materials

Meloxicam (MEL, 4-hydroxy-2-methyl-*N*-(5-methyl-2-thiazolyl)-2*H*-benzothiazine-3-carboxamide-1,1-dioxide) was donated by EGIS Pharmaceuticals Plc. (Budapest, Hungary) for research work. Human serum albumin (HSA, lyophilized powder, purity > 97%), fluorescein isothiocyanate-labelled HSA (FITC-HSA), Tween 80 (Tween), P407, disodium hydrogen phosphate (Na_2_HPO_4_), sodium dihydrogen phosphate (NaH_2_PO_4_), polar brain lipid extract, cholesterol, mucin from porcine stomach (Type III), and all reagents for cell line studies were purchased from Sigma Aldrich Co. Ltd. (Budapest, Hungary) if not indicated otherwise. Analytical grade solvents such as methanol, dimethyl sulfoxide (DMSO) and dodecane were purchased from Molar Chemicals (Budapest, Hungary). Sodium hyaluronate (NaHA, Mw = 1400 kDa) was obtained from Gedeon Richter Plc. (Budapest, Hungary). In all experiments, water was purified by the Millipore Milli-Q^®^ 140 Gradient Water Purification System.

### 2.2. Preparation of In Situ Gelling MEL-HSA Nanoparticle Formulations

**Figure 1:**
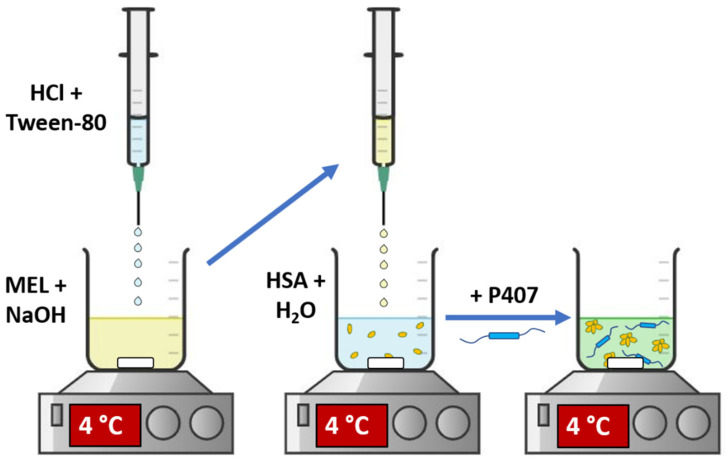
Preparation of MEL-HSA-P407.

MEL-HSA nanoparticles were produced by applying a modified coacervation method (Figure 1) according to the following steps [23]: first, Tween-80 was dispersed in 4 mL of HCl solution (0.1 M), whereas MEL was dissolved in 4 mL of NaOH solution (0.1 M) and HSA was dissolved in 8 mL of purified water. Then, the Tween 80 solution was added dropwise (0.5 mL/min) to the MEL solution at 4 °C under constant stirring (800 rpm). Next, this MEL–Tween 80 solution was added dropwise to the HSA solution at 4 °C under constant stirring (800 rpm). After complete homogenization, additional HCl was added dropwise to adjust pH to 5.6, and as a result the solution became turbid. Then, the formulation was incubated for 12 h under constant stirring (800 rpm) to obtain nanoparticle dispersion. P407 in various concentrations (based on preliminary experimental results 12, 13, 14, 15 and 16% *w*/*w*) was added to the formulation and kept in a cool place (5 ± 3 °C) overnight until complete hydration and dissolution of the polymer, before further investigations.

### 2.3. QbD-Based Comparative Risk Assessment

As the in situ thermo-gelling carrier system is part of a continuous development process, it needs to be compared to the HSA nanoparticles, validating the product life cycle, and therefore ensuring quality. At first, the quality target product profile (QTPP) was determined based on the mandatory requirements of a nose-to-brain applicable nanocarrier. The critical quality attributes (CQAs) were also determined as they are physicochemical factors affecting product safety, efficacy and quality. Critical process parameters (CPPs) and critical material attributes (CMAs) were not taken into account during the risk assessment process as an extensive optimization of P407 concentration was performed. For the identified elements, risk levels were assigned for both the MEL-HSA and MEL-HSA-P407. A three-level scale was used to describe the relation between these parameters: each relation was assigned with a “high” (H), “medium” (M) or “low” (L) attributive. To quantify the risk values, LeanQbD^®^ Software (QbD Works LLC, Fremont, CA, USA) was used. As the output of this comparative risk assessment, severity scores were compared and evaluated to determine the influence of the in situ thermo-gelling carrier system on product quality.

### 2.4. Optimization of In Situ Thermogelling Carrier System

#### 2.4.1. Rheological Studies

The rheological measurements were carried out with a Physica MCR302 rheometer (Anton Paar, Graz, Austria). A cone and plate type measuring device with cone angle of 1° was applied; the diameter of the cone was 25 mm, and the gap height in the middle of the cone was 0.046 mm. The gelation temperature was measured while the temperature was increased from 20 to 40 °C, using 1 °C/min heating rate. The measurement was performed at a constant frequency of 1.0 rad/min and at a constant strain of 1%. The gelation time of the polymer solutions was followed at a constant frequency of 1.0 rad/min and at a constant strain of 1% at 37 °C. The samples were stored at 5 ± 1 °C and taken immediately before the measurement. Viscoelastic character was determined by frequency sweep tests immediately after the gelation measurement, with a strain of 1% at 37 °C. Storage modulus (G’), loss modulus (G”) and loss factor were determined over the angular frequency range from 0.1 to 100 rad/s. The applied strain value (1%) was in the range of the linear viscoelasticity of the gels.

#### 2.4.2. Muco-Adhesion Measurement

Muco-adhesion was analyzed by means of tensile tests (TA-XT Plus texture analyzer (Metron Kft, Budapest, Hungary)) equipped with a 5-kg load cell. As a simulated mucosal membrane, a filter paper (Whatman^®^ qualitative filter paper, Sigma Aldrich Co. Ltd., Budapest, Hungary) with 25 mm diameter, impregnated with 50 µL of an 8% *w*/*w* mucin dispersion, was used, prepared with a simulated nasal electrolyte solution (SNES) consisting of 8.77 g/L sodium chloride (NaCl), 2.98 g/L potassium chloride (KCl), 0.59 g/L anhydrous calcium chloride (CaCl_2_) and dissolved in purified water; the pH was adjusted to 5.6 with 0.1 M HCl [12]. Five parallel measurements were performed. 20 mg of the sample was attached to the cylinder probe and placed in contact with the filter paper wetted with mucin. A 2500 mN preload was used for 3 min, then the cylinder probe was moved upwards to separate the sample from the substrate at a prefixed speed of 2.5 mm/min. The maximum detachment force (adhesive force) and the work of adhesion (A, mN/mm) were measured, the latter calculated as the area (AUC) under the ‘‘force versus distance’’ curve using the Exponent Connect software of the instrument. The formulations were thermostated at 37 °C for 30 min before measurement. As a reference system, 0.5% *w*/*w* NaHA aqueous solution was applied.

#### 2.4.3. Characterization of Nanoparticles

The formulations were characterized according to their average hydrodynamic diameter (Z-average), polydispersity index (PdI) and zeta potential using a Malvern Zeta sizer Nano ZS (Malvern Instruments, Worcestershire, UK) at 25 and 35 °C in folded capillary cells. The refractive index was set to 1.72. The pH of formulations was measured applying a WTW^®^ inoLab^®^ pH 7110 laboratory pH tester (Thermo Fisher Scientific, Budapest, Hungary). The osmolality of formulations was determined by osmometer (Knauer Semi-micro Osmometer, Berlin, Germany) based on the freezing point depression method. Each measurement was carried out in triplicate and data are shown as means ± SD. The encapsulation efficiency and loading capacity of gel-embedded nanoparticles were prepared with a Hermle Z323K high performance refrigerated centrifuge (Hermle AG, Gossheim, Germany) at 17.500 rpm, 4 °C for 30 min. The amount of free MEL in the supernatant was determined by high performance liquid chromatography (HPLC). Encapsulation efficiency (EE) and loading capacity (LC) of formulations were calculated according to the following equations [24]:

(1) $$EE\;\left(\%\right)=\frac{Amount\;of\;drug\;applied-Amount\;of\;drug\;in\;the\;supernatant}{Amount\;of\;drug\;applied}\cdot 100$$

(2) $$LC\;\left(\%\right)=\frac{Mass\;of\;drug\;encapsulated}{Mass\;of\;nanoparticles}\cdot 100$$

The distribution of FITC labelled HSA-MEL nanoparticles in gel structure was visualized by a Leica TCS SP5 confocal laser scanning microscope (Leica Microsystems GmbH, Wetzlar, Germany) and Visitron spinning disk confocal system (Visitron Systems GmbH, Puchheim, Germany). The P407 containing formulations and the FITC labelled HSA-MEL colloidal solution as reference were dropped onto slides and incubated for 10 min at 35 °C for thermo-gelling. Then, slides were excited with a 488 nm Argon laser, and fluorescence was detected with a 505 to 570 nm BP filter.

#### 2.4.4. Rapid Equilibrium Dialysis (RED)

In order to investigate the in vitro dissolution kinetics and release profile of different MEL-HSA-P407 formulations at nasal conditions, the RED Device (Thermo ScientificTM, Waltham, MA, USA) was used. A suspension of MEL was prepared in a phosphate buffer saline (PBS, pH 5.6) with a nominal concentration of 2 mg/mL as a control for the study. Both the control and in situ gelling MEL-HSA formulations were homogenized using an Eppendorf MixMate (Thermo Scientific^TM^, Waltham, MA, USA) vortex mixer for 30 s and an ultrasonic bath (Sonorex Digiplus, Bandelin GmbH & Co. KG, Berlin, Germany) for 10 min. The RED Device inserts (8K MWCO) were fitted into the PTFE base plate, then 150 µL of samples was placed into the donor chambers, while 300 µL of PBS (pH 5.6) was added to the acceptor chambers. Thereafter, the RED unit was covered with a sealing tape and incubated above gelling temperature (37 °C) on an orbital shaker (at 350 rpm) for 4 h. 50 μL aliquots were withdrawn from the acceptor chamber at 5, 15, 30, 60, 120 and 240 min time points and immediately replaced with the same amount of fresh medium. 50 μL of acetonitrile was added to the withdrawn samples and the MEL content was determined using HPLC. Five parallel measurements were performed.

#### 2.4.5. High Performance Liquid Chromatography (HPLC)

The determination of MEL concentration was performed with an Agilent 1260 HPLC (Agilent Technologies, Santa Clara, CA, USA). A Kinetex^®^ C18 column (5 µm, 150 mm × 4.6 mm (Phenomenex, Torrance, CA, USA)) was used as stationary phase. The mobile phases consisted of 0.065 M KH_2_PO_4_ aqueous solution adjusted to pH = 2.8 with phosphoric acid (A), and methanol (B). A linear gradient from 50–50% to 25–75% (A-B eluent) was applied from 0 to 14 min. Then, from 14 to 20 min the phase composition was set back to 50–50% A-B. Separation was performed at 30 °C with 1 mL/min flow rate. 10 µL of the samples was injected to determine the MEL’s concentration at 355 ± 4 nm using the UV-VIS diode array detector. Data were evaluated using ChemStation B.04.03. Software (Agilent Technologies, Santa Clara, CA, USA). The retention time of MEL was observed at 14.34 min. The regression coefficient (R^2^) of the calibration curve was 0.999 in the concentration range 1–200 μg/mL. The determined limits of detection (LOD) and quantification (LOQ) of MEL were 16 ppm and 49 ppm, respectively.

#### 2.4.6. In Vitro BBB Permeability Assay

Parallel artificial membrane permeability assay (PAMPA) was used to determine the brain specific effective permeability of MEL from the reference suspension and the MEL-HAS-P407 formulations [25]. The filter donor plate (Multiscreen™-IP, MAIPN4510, pore size 0.45 µm; Millipore, Merck Ltd., Budapest, Hungary) was coated with 5 µL of lipid solution containing 16 mg brain polar lipid extract (porcine) and 8 mg cholesterol dissolved in 600 µL dodecane. The Acceptor Plate (MSSACCEPTOR; Millipore, Merck Ltd., Budapest, Hungary) was filled with 300 μL of a PBS solution of pH 7.4. 150–150 μL of the formulation and the reference solutions were applied on the membrane of the donor plate. Then, this was covered with a plate lid in order to decrease the possible evaporation of the solvent. This sandwich system was incubated at 37 °C for 4 h (Heidolph Titramax 1000, Heidolph Instruments, Schwabach, Germany). The concentration of MEL permeated in the acceptor plate was determined using HPLC. The effective permeability and membrane retention of drugs were calculated using the following equation [25]:

(3) $$P_{e}\;\left(\mathrm{cm}/s\right)=-\frac{2.303\cdot V_{A}}{A\left(t-\tau_{SS}\right)}\cdot \log\left[1-\frac{c_{A}\left(t\right)}{S}\right]$$

where *P_e_* is the effective permeability coefficient (cm/s), *A* is the filter area (0.24 cm^2^), *V_A_* is the volume of the acceptor phase (0.3 mL), *t* is the incubation time (s), *τ_SS_* is the time to reach the steady state (s), *c_A_*(*t*) is the concentration of the compound in the acceptor phase at time point *t* (mol/mL), and *S* (mol/mL) is the solubility of MEL in the donor phase. The latter was determined after centrifugation (at 12000 rpm, 15 min, Eppendorf Centrifuge 5804 R) in Microcon Centrifugal Filter Devices (30,000 MWCO) and 50-times dilution of the formulations, using the same HPLC system. The flux of samples was calculated using the following equation [26]:

(4) $$Flux\;(\mathrm{mol}/{\mathrm{cm}}^{2}\cdot s)=P_{e}\cdot S$$

### 2.5. Cell Line Studies with Optimized Formulation

#### 2.5.1. Cell Cultures

Human RPMI 2650 (ATCC cat. no. CCL 30) nasal epithelial cells were grown in Dulbecco’s Modified Eagle’s Medium (DMEM, Gibco, Life Technologies, Gaithersburg, MD, USA) supplemented with 10% *v*/*v* fetal bovine serum (FBS, Pan-Biotech GmbH, Aidenbach, Germany) and 50 µg/mL gentamicin in a humidified 37 °C incubator with 5% CO_2_. The surfaces were coated with 0.05% rat tail collagen in sterile distilled water before cell seeding in culture dishes and the medium was changed every 2 days. When RPMI 2650 cells reached approximately 80–90% confluence in the dish, they were trypsinized with 0.05% trypsin-0.02% EDTA solution. One day before the experiment, retinoic acid (10 µM) and hydrocortisone (500 nM) were added to the cells to form a tighter barrier [27].

For permeability measurements epithelial cells were co-cultured with human vascular endothelial cells [28,29] to create a more physiological barrier, representing both the nasal epithelium and the submucosal vascular endothelium. The endothelial cells were grown in endothelial culture medium (ECM-NG, Sciencell Research Laboratories, Carlsbad, CA, USA) supplemented with 5% FBS, 1% endothelial growth supplement (ECGS, Sciencell Research Laboratories, Carlsbad, CA, USA) and 0.5% gentamicin on 0.2% gelatin-coated culture dishes (10 cm). For the permeability experiments, cells were used at passage 8.

#### 2.5.2. Cell Viability Measurements

Real-time cell electronic sensing is a non-invasive, label-free, impedance-based technique to quantify the kinetics of proliferation and viability of adherent cells. Our group has successfully used this method to study cell damage and/or protection in living cells [30,31]. The RTCA-SP instrument (ACEA Biosciences, San Diego, CA, USA) monitored the impedance of cell layers every 10 min. Cell index was defined as R_n_-R_b_ at each time point of measurement, where R_n_ is the cell-electrode impedance of the well when it contains cells, and R_b_ is the background impedance of the well with the medium alone. Cell index values reflect cell number and viability.

The 96-well E-plates with integrated gold electrodes (E-plate 96, ACEA Biosciences, USA) were coated with 0.2% gelatin and incubated for 20 min in the incubator. Then gelatin was removed, and culture medium (50 μL) was added to each well for background readings. RPMI 2650 cell suspension was dispensed at the density of 2 × 10^4^ cells/well in 50 µL volume and the plate was kept in a humidified incubator with 5% CO_2_ at 37 °C. When cells reached a steady growth phase, they were treated with the nano-formulations and their components.

#### 2.5.3. Permeability Study on the Co-Cultured Model

The tightness of the BBB co-culture model was verified by transepithelial electric resistance (TEER) measurement, which reflects the tightness of cell layers of biological barriers. TEER was measured by an EVOM volt-ohm meter (World Precision Instruments, Sarasota, FL, USA) combined with STX-2 electrodes, and it was expressed relative to the surface area of the monolayers as Ω × cm^2^. TEER of coated, but cell-free, filters were subtracted from measured TEER values. Cells were treated with the nano-formulations when the cell layer reached steady TEER values.

For the permeability studies we used a co-culture model, in which RPMI 2650 cells were cultured together with endothelial cells on inserts of a 12-well trans-well system (Transwell, polycarbonate membrane, 3 µm pore size, 1.12 cm^2^, Corning Costar Co., Lowell, MA, USA) for 5 days. In this model, endothelial cells were seeded (1 × 10^5^ cells/cm^2^) to the bottom side of culture inserts coated with Matrigel (growth factor reduced, BD Biosciences, San Jose, CA, USA) and nasal epithelial cells were passaged (2 × 10^5^ cells/cm^2^) to the upper side of the membranes coated with rat tail collagen.

During the permeability experiments, the inserts were placed on 12-well plates containing 1.5 mL Ringer-HEPES buffer in the acceptor (lower/basal) compartments. In the donor (upper/apical) compartments, the culture medium was changed and 0.5 mL buffer containing different formulations and meloxicam as reference were added. To avoid an unstirred water layer effect, the plates were kept on a horizontal shaker (120 rpm) during the assay in a humidified incubator with 5% CO_2_ at 37 °C for 1 h. After incubation, samples were collected from the donor and acceptor compartments and the meloxicam concentration was measured by HPLC.

To test the function of our co-culture model, the flux of permeability marker molecules FITC-labeled dextran (FD10, Mw: 10 kDa) and Evans blue labeled albumin (EBA; MW: 67.5 kDa) was determined across the cell layers [31]. In the donor compartments of the inserts, 0.5 mL buffer containing FD10 (100 μg/mL) and EBA (167.5 μg/mL Evans blue dye and 10 mg/mL bovine serum albumin) was added and 12-well plates were placed on a horizontal shaker (120 rpm) for 30 min. After treatments, samples from the lower compartments were collected and the markers were measured with a fluorescence multi-well plate reader (Fluostar Optima, BMG Labtech, Ortenberg, Germany; FITC: excitation wavelength: 485 nm, emission wavelength: 520 nm; Evans-blue labeled albumin: excitation wavelength: 584 nm, emission wavelength: 680 nm).

The apparent permeability coefficients (*P_app_*) were calculated as described previously [31] by the following equation:

(5) $$P_{app}(\mathrm{cm}/s)=\frac{\Delta {\left[C\right]}_{A}\times V_{A}}{A\times {\left[C\right]}_{D}\times \Delta t}$$

Briefly, *P_app_* was calculated from the concentration difference of the tracer in the acceptor compartment (Δ[*C*]*_A_*) after 60 min, initial donor compartments’ concentration ([*C*]*_D_*), *V_A_* is the volume of the acceptor compartment (1.5 mL) and *A* is the surface area available for permeability (1.1 cm^2^).

#### 2.5.4. Treatments of Cultured Cells

For cell viability measurements, 10, 30 and 100 times dilutions from optimized MEL-HSA P407 formulation (14% *w*/*w* P407), 2 mg/mL meloxicam, 3 mg/mL Tween 80 and 160 mg/mL P407 solutions were prepared in cell culture medium. For permeability measurements, 2 mg/mL meloxicam and 10× times dilution of MEL-HSA-P407 were prepared in Ringer-HEPES buffer and added to the donor compartments.

#### 2.5.5. Immunohistochemistry

To evaluate morphological changes in RPMI 2650 cells caused by the MEL-HAS-P407 formulation and MEL, immunostaining for junctional proteins zonula occludens protein-1 (ZO-1) and β-catenin was made. After the permeability experiments, cells on culture inserts were washed with phosphate buffer (PBS) and fixed with ice cold methanol–acetone (1:1) solution for 2 min then washed with PBS 3 times. The nonspecific binding sites were blocked with 3% bovine serum albumin in PBS. Primary antibodies rabbit anti-ZO-1 (AB_138452, 1:400; Life Technologies, Carlsbad, CA, USA) and rabbit anti-β-catenin (AB_476831, 1:400) were applied as overnight treatment at 4 °C. Incubation with anti-rabbit IgG Cy3 conjugated (AB_258792, 1:400) secondary antibodies lasted for 1 h and Hoechst dye 33342 was used to stain cell nuclei. After mounting the samples (Fluoromount-G; Southern Biotech, Birmingham, AL, USA) staining was visualized by Leica TCS SP5 confocal laser scanning microscope (Leica Microsystems GmbH, Wetzlar, Germany).

### 2.6. Statistical Analysis

All data presented are means ± SD. In the case of mucoadhesive study, an unpaired *t*-test was applied. The values in the cell line studies were compared using the analysis of variance followed by Dunett or Bonferroni tests using GraphPad Prism 5.0 software (GraphPad Software Inc., San Diego, CA, USA). Changes were considered statistically significant at *p* < 0.05. The significance of differences of RED and PAMPA data was calculated with one-way ANOVA with post hoc test (Tukey’s multiple comparisons test, α = 0.05).

## 3. Results

### 3.1. Comparative Risk Assessment of MEL-HSA and MEL-HSA-P407

In addition to CQAs, well-defined goals must be set for the product to fit the nose-to-brain route of administration. This route has many obstacles and challenges to overcome, starting from the formulation process and the patient’s administration until the developed effect in the central nervous system. Due to the physiological and chemical variety through this pathway, a number of formulation aspects must be taken into consideration ensuring the desired product quality and performance. This is determined by the QTPP of the product, the elements of which can be seen in Table 1.

**Figure 2:**
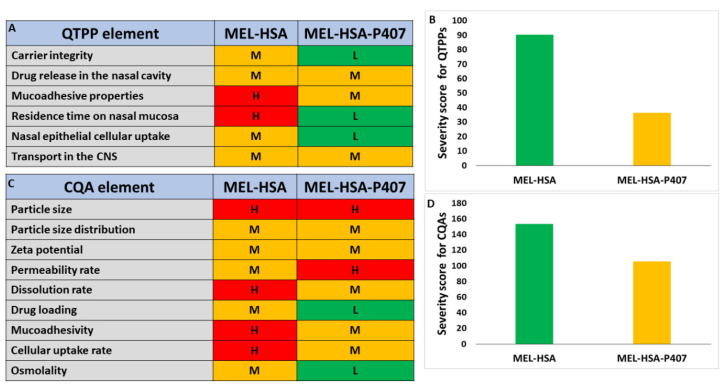
Interdependence rating amongst QTPPs (**A**), CQAs (**C**) and MEL-HSA, MEL-HSA-P407 formulations with the corresponding severity scores for QTPPs (**B**) and CQAs (**D**). Abbreviations: H: high, L: low, M: medium.

The next step was to assign risk relations to MEL-HSA and MEL-HSA-P407 formulations which were first prepared by interdependence rating on a three-level scale. Quantifying these risks was interpreted using severity scores which were compared to each other. The tables of the interdependence rating and the severity scores are shown in Figure 2.

Based on the calculated severity scores, it can be claimed that, by incorporating HSA nanoparticles as a part of an in situ thermo-gelling system, the severity of risk can be heavily reduced. As desired for the nose-to-brain pathway, in theory, with increased muco-adhesion properties, an increased residence time can be observed. The particle characteristics are always of paramount importance and cannot be left out of the design space development. These parameters are closely related to the dissolution and absorption profile of the nanocarrier. The reduction in the added-up severity score means that the product quality can be improved and is the reason for the continuous manufacturing improvement. With optimal P407 concentration, the increased residence time and muco-adhesivity along with the penetration-enhancement also contribute to the possibly increased drug release and permeability. However, individual risks must be investigated, as seen from the structure of this article. The risk of decrease in permeability due to the more structured gel system, with less API exposed to the dissolution media per time, is always there, which is why it is of the utmost importance and can be characterized as a high-risk relation. This possible unbeneficial risk can be countered by the improved residence behavior in the nasal cavity, providing the pharmacokinetics to an adequate extent. P407 itself has also a solubilizing effect which also opposes some of the unbeneficial possibilities. The determination of gelling properties also helps to evaluate the success of the development, so these factors are also investigated; however, this cannot be part of a comparative risk assessment, as MEL-HSA nanoparticles themselves do not contain any gelling material.

### 3.2. Optimization of P407 Concentration in the Formulation

#### 3.2.1. Investigation of In Situ Thermo-Gelling Properties

**Figure 3:**
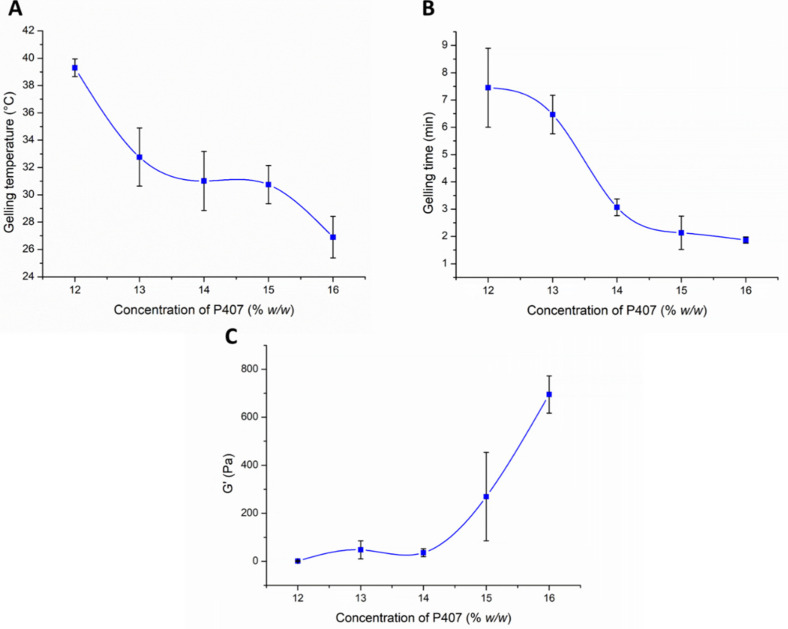
Effect of the P407 concentration on the gelling temperature (**A**), gelling time at 37 °C (**B**), and gel strength (**C**). Data is presented as means ± SD, *n* = 5.

During the optimization process, the effect of the P407 concentration on the gelation and rheological properties was analyzed. In the first part of our rheological investigation, the effect of the P407 concentration on the lower critical solution temperature (LCST) was measured. LCST was defined as the temperature at the crossover point of the storage modulus (G’) and loss modulus (G”), and primary data can be seen in Figure S1. Based on our results, we can conclude that a minimum of 13% *w*/*w* P407 concentration is needed for gelling at body temperature (Figure 3A). However, considering possible dilution in vivo, it is preferable to use a slightly higher concentration (at least 14% *w*/*w* or above).

The gelling time significantly affects the bioavailability of the nasal preparation. If gelation is too slow, the mucosal concentration of the product is significantly reduced due to the elimination mechanisms, and dilution with a liquid layer covering the mucosa prevents subsequent gelation. On the other hand, too fast a gelation can make it difficult to distribute the composition on the mucosa, therefore a reduced absorption surface can be expected. It can be clearly seen from our results that in case of 12–13% *w*/*w* P407 gelation time was too slow (6–7 min), but with the increasing polymer concentration (14–16% *w*/*w*) gel formation occurs within 2–3 min at 37 °C (Figure 3B), which seems to be optimal for nasal application.

The rheological properties of gels at body temperature may also affect the bioavailability of the formulations. The stronger the gel structure, the more resistant it is against the mucosal elimination mechanisms. The nasal mucosa is characterized by the phenomenon of mucociliary clearance caused by the beating of the cilia. The effect of ciliary beating can usually be simulated by oscillatory rheological measurements at low oscillation frequency [32]. In our case, we compared our formulations at the angular frequency of 1 rad/s using frequency sweep tests.

As can be seen in Figure 3C an elastic characteristic can be measured with only higher P407 concentrations, i.e., only samples containing 15–16% *w*/*w* P407 can be characterized with considerable elasticity at body temperature. Although gelation begins at body temperature in the case of the 13–14% *w*/*w* systems, this results in only a very weak gel structure, which may not hinder drug release. As was also described in the literature [33], the poloxamer forms weak gels.

#### 3.2.2. Muco-Adhesion Measurement

Gelling at body temperature results in the micellar arrangement of P407 molecules, which may affect the muco-adhesiveness of the formulation. During the tensile test, the contact of the two surfaces can generate the change in the gel structure and the formation of the mucoadhesive interaction simultaneously. As a result of the two processes, we can observe high variability in the adhesive work and force values.

**Figure 4:**
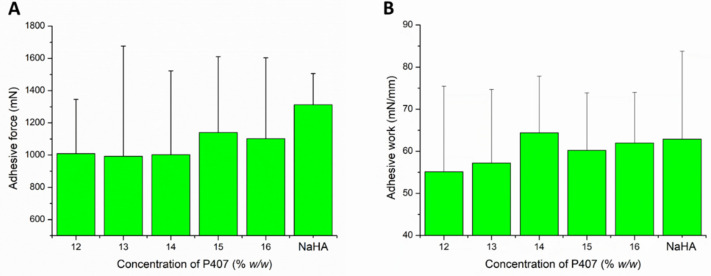
Adhesive force (**A**) and adhesive work (**B**) of the compositions in various P407 concentrations. Data is presented as means ± SD, *n* = 5.

In the case of our systems, adhesive forces of about 1000 mN were measured in almost all cases (Figure 4A), and it was found that the mucoadhesive force did not change remarkably with increasing P407 concentration. Only a slightly increasing tendency can be observed at higher polymer concentration (1100 mN). This can mean that the number of functional groups forming mucoadhesive bonds at the interface slightly increases. Comparing the muco-adhesivity of the in situ gelling systems with that of the NaHA, we can see a higher adhesive force value in the case of NaHA, but the difference cannot be considered as significant (*p* > 0.05 in each cases).

In contrast, there is a slight increase in mucoadhesive work, and higher values of adhesive work can be measured in those compositions (14–16% *w*/*w* P407) where a gel structure is already formed at near body temperature (Figure 4B). In this case, with the formation of the gel structure, the formation of mucoadhesive physical bonds is more significant. Comparing the formulated systems with the reference mucoadhesive, we could not detect significant differences (*p* > 0.05 in each cases), which could be the result of the large SD.

#### 3.2.3. Characterization of Carrier Systems

Nanoparticle properties such as particle size, PdI, zeta potential, encapsulation efficacy, loading capacity, pH and osmolality are key parameters for characterization of the therapeutic applicability of nasal formulations. These nasal administration-related parameters of the P407-containing formulation in different concentrations are presented in Table 2.

**Figure 5:**
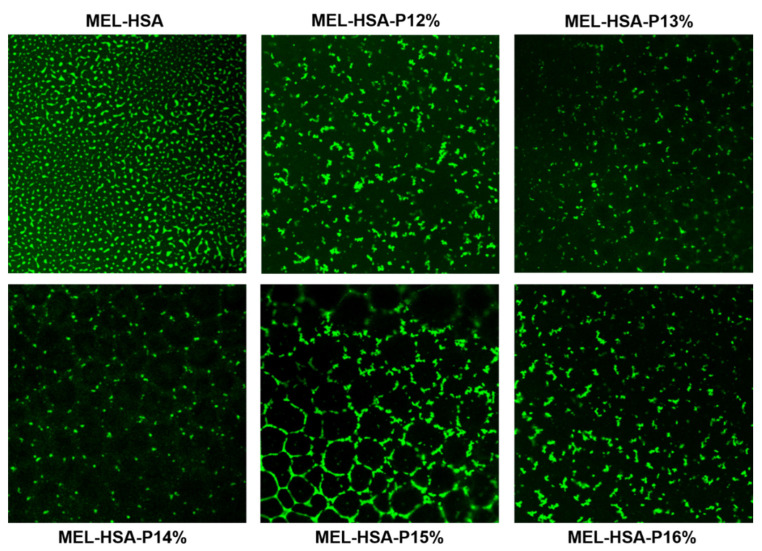
FITC-labelled MEL-HSA nanoparticles and their distribution in gel structure containing various concentrations of P407 at 60× magnification.

The Z-average, PdI and zeta potential value of formulations was measured both in sol and gel state. Formulations containing 12–14% *w*/*w* P407 meet the requirements of intranasally administered nanoparticles (<200 nm) even after thermo-gelling, but in case of 15–16% *w*/*w* P407 these parameters are out of the acceptance criteria. Moreover, the Z-average (in the range of 180–193 nm) of gel embedded MEL-HSA nanoparticle suggests increased drug release. The narrow PdI (from 0.193 to 0.328) indicates monodispersed size distribution, which ensures the homogeneity of nanoparticles in the gel structure. The distribution of FITC labelled MEL-HSA nanoparticles was visualized in the gel structure using fluorescent microscopy (Figure 5).

The fluorescent, microscopic images prove the homogenous distribution of MEL-HSA nanoparticles and narrow PdI, and gelling of the formulations did not show an aggregation tendency of the nanoparticles. There was no remarkable difference in zeta potential (between −9.4 and −7.0 mV) of formulations and these values indicate that repulsion of nanoparticles is not significant enough to avoid aggregation in sol form. Both encapsulation efficacy and loading capacity of formulations were slightly decreased with increasing P407 concentration, but even so this difference was not remarkable, therefore from this point of view each formulation can be suitable for application. The osmolality of formulations containing 12–15% *w*/*w* P407 was hypotonic (200–278 mOsmol/L), which predicts enhanced drug absorption, offsetting the retention of gel structure. In case of 16% *w*/*w* P407 the formulation was hypertonic (311 mOsmol/L), which can result in significant dehydration of nasal mucosa; therefore, from a therapeutic point of view the application of MEL-HSA-P16% is not recommended.

#### 3.2.4. In Vitro Dissolution Profiles (RED)

**Figure 6:**
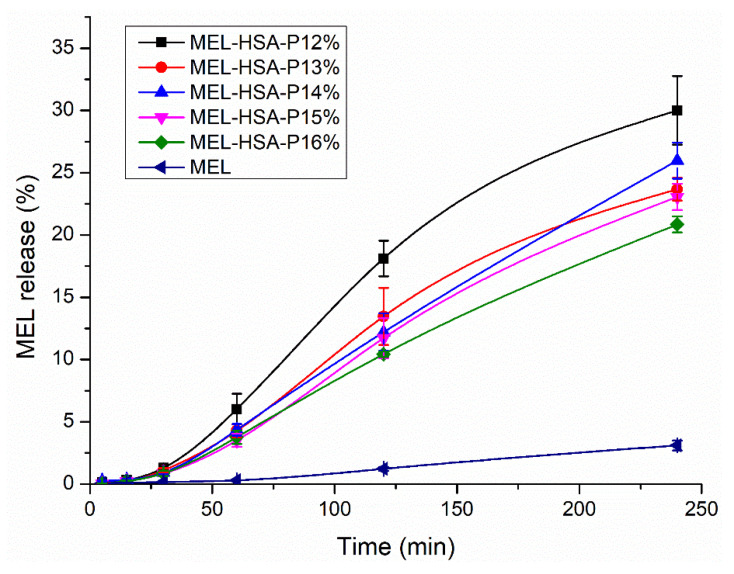
In vitro dissolution profiles of MEL-HSA-P407 formulations in comparison to starting MEL. Data is presented as means ± SD, *n* = 5.

The drug release kinetic of in situ gelling formulations is a critical part of rational drug design as it is a major determinant of the efficacy of delivery of the carrier in vivo and the subsequent release of the free drug. The in vitro release profile reveals important information on the structure and behavior of the formulation, on possible interactions between the drug and carrier composition, and on their influence on the rate and mechanism of drug release. The dialysis-based release method is a well-established and useful technique to study in vitro release from nano-particulate delivery systems. RED device was developed in order to reduce the time to equilibrium, and to provide results faster than other dialysis methods [34]. In this system, MEL conjugated HSA nanoparticles are physically separated from the acceptor media by a dialysis membrane with 8 kDa cutoff which allows only the passive diffusion of free MEL into the acceptor media. The time-dependent in vitro release profiles of MEL and formulations were determined with RED (Figure 6).

The in vitro dissolution profiles of MEL-HSA-P407 and starting MEL were investigated in intranasal conditions (pH = 5.6). Due to the weak acidic character of MEL (*pK_a_* = 3.43 [26]) it can be found in fully ionized form in dissolution medium ($2<\Delta pH=\left|pH-pK_{a}\right|)$, although the dissolution profile of pure MEL shows only a slight increase. In the case of formulations, a significantly increased dissolution was observed compared to pure MEL (**, *p* < 0.01), due to the nano size and increased specific surface area of MEL-HSA nanoparticles and the solubilizing effect of Tween. The dissolution profiles of the formulations clearly demonstrate the effect of polymer on drug release. When increasing the concentration of P407 in the formulation, the dissolution of MEL was slower, which can be explained by the hindered liberation of MEL through the more viscous gel structure. Interestingly, formulations containing P407 in a lower concentration (12–13% *w*/*w*) followed Hixon-Crowell kinetics, which is presumably the less effective anti-aggregation effect due to the reduced polymer concentration; but in higher concentrations (14–16% *w*/*w*) zero-order kinetics dominated (Table 3) [35]. The best fit of kinetic data is presented in Figure S2. Similar types of effect of P407 gel on the release behavior of insulin [36], paclitaxel [37], ceftiofur [38] and recombinant hirudin [39] have also been observed, in which the mode of drug release followed zero order.

#### 3.2.5. In Vitro BBB Permeability

**Figure 7:**
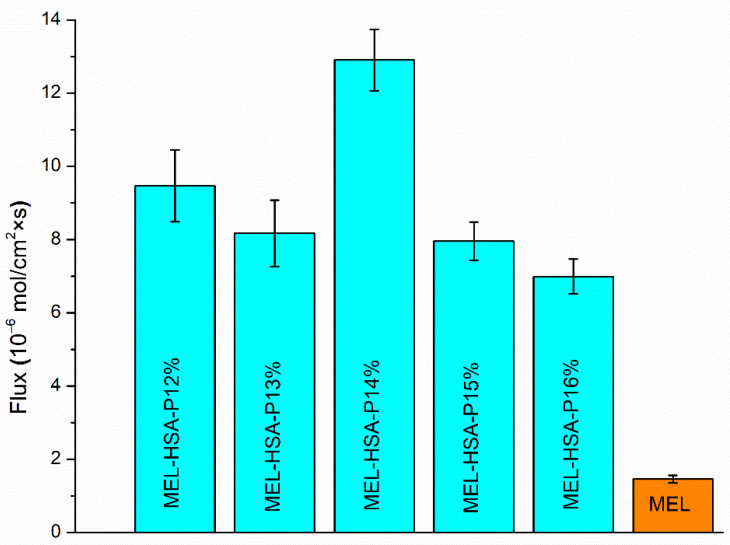
Fluxes in PAMPA-BBB permeability study of MEL-HSA-P407 formulations compared to starting MEL. Data is presented as means ± SD, *n* = 6.

In the case of nasal administration, it should be taken into account that a fraction of the drug can be absorbed into the systemic circulation, from where it can only reach the brain if it is able to pass the BBB. It has been reported that NSAIDs have low disposition in the CNS via systemic circulation [40]. To investigate this formulation ability, the brain lipid-specific PAMPA permeability assay (PAMPA-BBB) is suitable. The PAMPA-BBB results of MEL-HSA formulations containing P407 in various concentrations in comparison to pure MEL are shown in Figure 7. Each P407 containing formulation showed significantly higher flux compared to pure MEL (**, *p* < 0.01), which can be explained by the enhanced solubility of MEL due to the solubilizing effect of HSA and P407. Moreover, in the case of 14% *w*/*w* P407 the flux was by far the highest among all of the formulations (**, *p* < 0.01). This phenomenon can be explained by the adequate balance of the resultant effect of nanoparticle characteristics and drug release kinetics. Formulations containing P407 in lower concentrations (12–13%) followed Hixon-Crowell kinetics, assuming a lower sustained saturation of drug, whereas the formulations containing P407 in higher concentrations (14–16%) followed zero order kinetics, indicating a faster drug release. From these zero-order kinetics, the following compositions MEL-HSA-P14% had the highest rate constant (0.368 µg min^−1^), which can be rationalized as the smaller Z-average resulting in a higher specific surface area; therefore, the higher dissolution rate ensures an increased gradient between donor and acceptor phases promoting permeation. In case of lower polymer concentration (12–13%) the effect of different drug release kinetics is negligible, as the remarkable Z-average difference of gel-embedded nanoparticles has a dominant influence on concentration in the donor phase.

Summing up, and based on the rheological, muco-adhesion, drug release and permeability studies, the 14% *w*/*w* P407 containing formulation (MEL-HSA-P14%) was considered as the optimized formulation from a therapeutic point of view. MEL-HSA-P14% forms a weak but mucoadhesive gel structure at 32 °C within 3 min which allows enhanced permeability of MEL through blood–brain barrier-specific lipids. Therefore, in the cell line studies, we decided to investigate that composition further.

### 3.3. Evaluation of Cell Line Studies

#### 3.3.1. Cell Viability Assay

**Figure 8:**
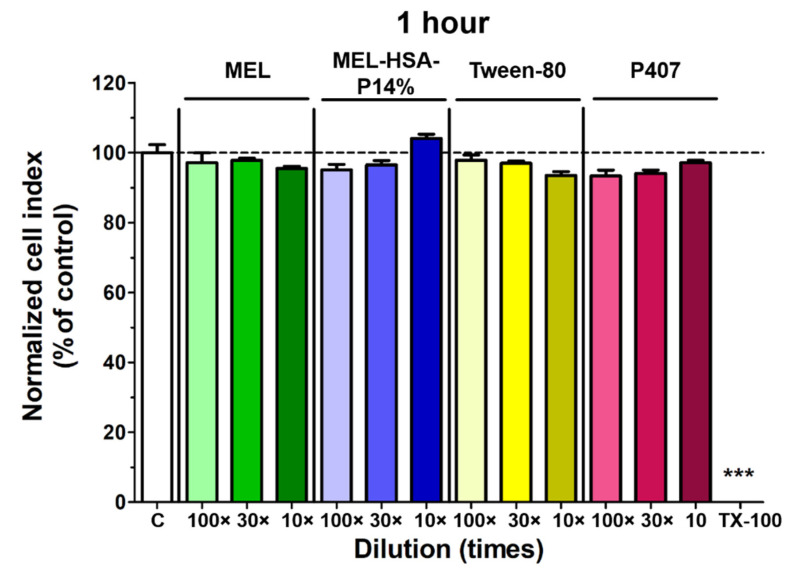
Cell viability of RPMI 2650nasal epithelial cells after a 1-h treatment with MEL, MEL-HSA-P14% formulation, or with their components, measured by impedance. The values are presented as a percentage of the control group (means ± SD, *n* = 6–12). Statistical analysis: ANOVA and Dunett’s test. *** *p* < 0.01, compared to the control group. TX-100, Triton X-100 detergent.

Impedance measurement as a sensitive method to detect alteration in cellular viability did not show cell damage after a 1-h treatment with MEL-HSA-P14% formulation or its components (Figure 8). As a comparison, the reference compound Triton X-100 detergent caused cell death, as reflected by the decrease in impedance.

#### 3.3.2. MEL Permeability across the Culture Model of the Nasal Mucosa Barrier

**Figure 9:**
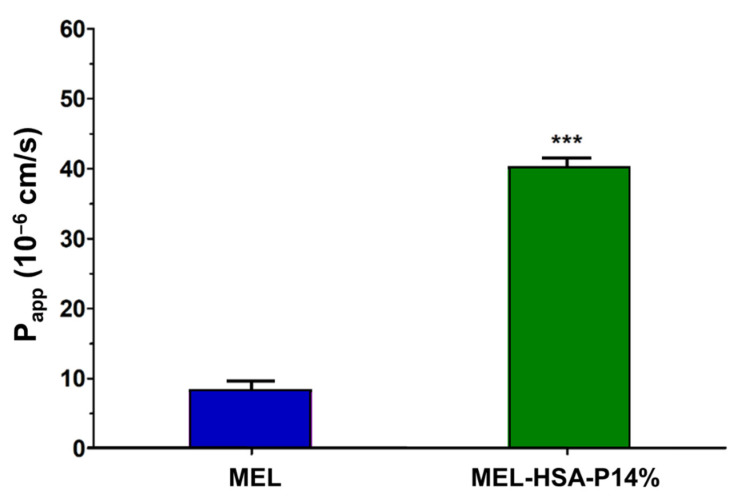
Permeability of MEL (2 mg/mL in all samples) and MEL-HSA-P14% nano-formulation across a co-culture model of human RPMI 2650 nasal epithelial cells and vascular endothelial cells (1-h assay). Values are presented as means ± SD, *n* = 3. *** *p* < 0.001 significantly different from MEL control.

The permeability of MEL was tested on the nasal epithelial and vascular endothelial cell co-culture model (Figure 9). After a 1-h treatment MEL in MEL-HSA-P14% showed significantly higher (four-fold) *P_app_* value compared with MEL suspension (MEL) (Figure 9). This result is in agreement with our previous finding using nanonized MEL in nasal formulation in the human RPMI 2650 nasal epithelial cell model [27] and also with results of PAMPA-BBB in the present study.

**Figure 10:**
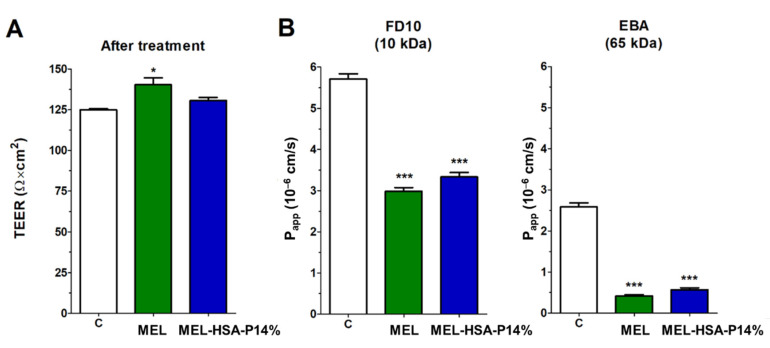
Transepithelial electrical resistance (TEER) of the co-culture model after a 1-h treatment with MEL and MEL-HSA-P14% (**A**). Values for paracellular permeability markers fluorescein-labeled dextran (FD10) and Evans blue-labeled albumin (EBA) after a 1-h treatment with MEL and the nano-formulation (**B**). Values are presented as means ± SD, *n* = 3. C: control; MEL; MEL-HSA-P14%. * *p* < 0.05; *** *p* < 0.001 significantly different from control.

We found adequate TEER and low permeability values for paracellular markers indicating a good barrier property of the nasal mucosa co-culture model (Figure 10). Both the TEER and *P_app_* values were better than in the mono-culture model we described earlier [27]. The TEER values of the co-culture model remained at the level of the control group after a 1-h treatment with MEL-HSA-P14%, indicating that it did not damage the barrier function of the model (Figure 10A). MEL increased TEER and both treatments decreased *P_app_* values for the two hydrophilic paracellular marker molecules, dextran and albumin (Figure 10B). This result suggests that the anti-inflammatory MEL may have a beneficial effect on the nasal barrier either alone or in formulation.

#### 3.3.3. Immunohistochemistry

**Figure 11:**
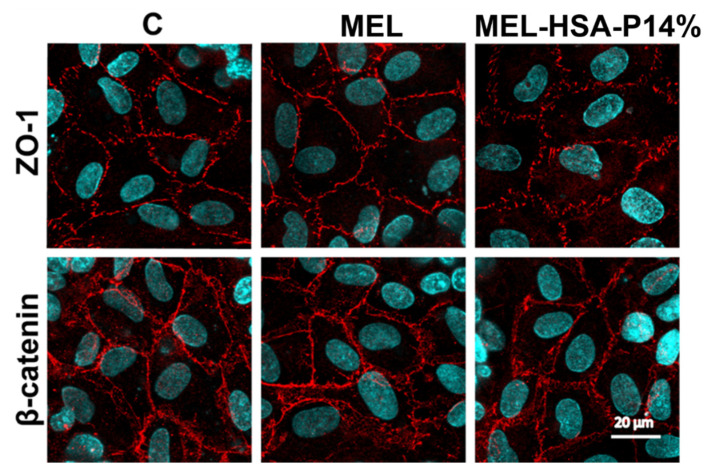
Immunostaining for junctional linker proteins ZO-1 and β-catenin on human RPMI 2650 nasal epithelial cell layers following a 1-h treatment with MEL and MEL-HSA-P14%. The control group (C) received only medium. Red: junctional proteins; blue: cell nuclei. Scale bar: 20 μm.

The staining pattern of the junctional linker proteins ZO-1 and β-catenin on human RPMI 2650 nasal epithelial cells in the co-culture model was typical for epithelial barriers. The cell shape was cobblestone, and the junctional proteins were visible in a belt-like manner at the cell borders (Figure 11), in concordance with our previous results [27]. We found no change in the staining pattern after the 1-h treatments as compared to the control group (Figure 11) indicating that the formulation did not damage the barrier model.

## 4. Discussion

A series of NSAIDs, including MEL, show significant potential in Alzheimer’s disease although their administration through conventional routes results in low deposition in the CNS due to severe elimination mechanisms and low capability of by-passing the BBB. Therefore, the application of a suitable nano carrier can be promising in increasing, their bioavailability [40]. HSA, as a versatile, biodegradable nano-carrier is an auspicious tool for intranasal delivery of NSAIDs, as several studies have already proved the successful nose-to-brain delivery of candidates like flurbiprofen [41] or MEL [13] itself. Although the exact pathway of HSA through nose-to-brain delivery is still not clearly demonstrated, experimental results showed that isotope labelled albumin could reach the brain after nasal administration [42].

The intranasal applicability of MEL-HSA nanoparticles is limited by mucociliary clearance, therefore application of the in situ gelling mucoadhesive polymer P407 can be advantageous, since the above mentioned LCST, PPO chains become less soluble resulting in micelle formation and entanglements followed by gelation of the sol form [43,44]. Several studies have demonstrated that poloxamer-based in situ gelling formulations have been successfully applied in numerous therapeutic fields especially among neurological diseases such as in the therapy of migraine [45], Parkinson’s syndrome [46], Alzheimer’s disease [47,48,49] and depression [50]. The basic ingredient of the therapeutic effectiveness of in situ thermo-gelling nasal formulations is their advantageous nature, which besides improving the pharmacokinetic profile of the administered drug also increases patients’ compliance [51]. Their sol form allows accurate dosing e.g., by the use of a metered dose nasal actuator system and after gelling, post-nasal drip into the throat can be reduced, therefore minimizing any undesired taste problem and loss of drug from the nasal cavity. The gel form ensures increased residence time of the formulation on nasal mucosa, exhibiting sustained release and supporting drug absorption. Moreover, gels can be used alongside demulcents or emollients which may not be suitable for solutions, suspensions or even powder dosage forms, in order to reduce the irritation potential.

Besides these mucoadhesive properties, particle characteristics play an important role in the applicability of formulations through the nasal administration route. It has been reported that nanoparticles with an average hydrodynamic diameter of 20–200 nm are the most suitable for nose-to-brain transport [52]. The PdI also plays a very important role in drug pharmacokinetics since a lower value indicates an enhanced probability of a more uniform absorption through the nasal mucosa, while a higher value may lead to pharmacokinetic irregularity and variability in the therapeutic outcome [53]. It is usually recommended that the PdI should be below 0.5. The zeta potential, which is dependent on both the surface charge of the particles and the ionic strength of the medium used for particle dispersion, is recommended to have a slightly negative or even positive surface charge, as this enhances nasal absorption as the nasal mucosa itself is negatively charged. Moreover, increased encapsulation efficacy and drug loading of nanoparticles is required to ensure proper dosing restricted by lower administration volume, up to a maximum of 200 µL. Our albumin nanoparticle gelling formulations containing 12–14% *w*/*w* P407 met these requirements and became suitable for intranasal administration after thermo-gelling; moreover, these parameters were more promising than in case of flurbiprofen-albumin nanoparticles, which were already successfully administered intranasally, directly to the brain, by Wong et al. [41].

Besides the particle characteristics of albumin nano-particles, the physiological properties of the nasal cavity should be also taken into account. The pH of the formulations was in the range of the human nasal mucosa’s physiologic pH range (4.62 to 7.00) supporting normal ciliary function and reducing the chance of damaging the barrier integrity of the nasal epithelial cells [54]. In this slightly acidic environment, lysozyme, the natural antimicrobial agent in the nose, is effective in the prevention of the growth of pathogenic bacteria in the nasal passage; therefore, preservative agents are negligible, which is usually incompatible with the applied polymer. Nasal preparations are normally isotonic (about 290 mOsmol/L), which is also best tolerated, but sometimes a deviation from isotonicity may be advantageous. Especially, hypotonic solutions can facilitate drug absorption through interfering with cilia movement [55]. Based on these perspectives, our optimized formulation MEL-HSA-P14% met all the listed requirements, which suggests suitable therapeutic applicability, which can be corroborated with the experimental results of Giuliano et al., according to which prolonged delivery of the drug for up to 5 h from P407 hydrogel was achieved via increasing the permeation profile of the compound, as measured through ex vivo porcine nasal mucosa [56].

## 5. Conclusions

In summary we optimized an in situ thermo-gelling MEL-HSA-P407 formulation, which can be administered in liquid state into the nostril. Temperature dependent sol-gel transition on the mucosa, resulting in increased resistance against mucoliciary clearance, can estimate improved drug absorption. Based on rheological, muco-adhesion, drug release and permeability studies, MEL-HSA-P14% was considered as the optimized formulation. Cell line studies showed no cell damage after a 1-h treatment with MEL-HSA-P14% on RPMI 2650 human endothelial cells; moreover, enhanced permeation (four-fold) of MEL from MEL-HSA-P14% was observed in comparison to MEL suspension. Overall, MEL-HSA-P14% is promising in terms of overcoming the challenges of nasal drug delivery by increasing resistance against mucociliary clearance, and therefore can improve nasal drug absorption.

## Figures and Tables

**Table 1:** QTPP elements of the nose-to-brain applicable HSA nanoparticle- and poloxamer-based in situ thermogelling system.

QTPP Element	Target
Carrier integrity	Nanosized particle size and distribution with uniform API content after gelation at gelation temperature.
Drug release in the nasal cavity	The formulation should release more MEL in the dissolution medium compared to initial MEL at pH 5.6.
Mucoadhesive properties	The mucoadhesive force and work should be high enough to meet the requirements of the nasal delivery.
Residence time on nasal mucosa	Increased residence time compared to non-gel formulations.
Nasal epithelial cellular uptake	The formulation should have higher permeability on nasal epithelial cells without damaging the cells forming the nasal barrier compared to raw MEL.
Transport in the central nervous system	The flux and permeability value of the gel formulation should be increased across BBB lipids compared to initial MEL

**Table 2:** Physico-chemical parameters of in situ thermogelling nasal formulations.

Notation of Formulation	Content of P407 (% *w*/*w*)	25 °C		35 °C		ZP (mV)	EE (%)	LC (%)	pH	Osmolality (mOsmol/kg)
		Z-Average (nm)	PdI	Z-Average (nm)	PdI					
MEL-HSA-P12%	12	172 ± 2	0.189 ± 0.02	180.7 ± 3	0.193 ± 0.03	−9.4 ± 0.7	82.34 ± 0.12	1.26 ± 0.01	5.85 ± 0.08	200 ± 2
MEL-HSA-P13%	13	175 ± 2	0.211 ± 0.01	188.7 ± 2	0.282 ± 0.02	−8.5 ± 0.5	82.01 ± 0.19	1.17 ± 0.02	5.81 ± 0.04	220 ± 3
MEL-HSA-P14%	14	176 ± 3	0.205 ± 0.02	193.7 ± 2	0.211 ± 0.02	−7.9 ± 0.3	81.64 ± 0.21	1.09 ± 0.02	5.61 ± 0.05	242 ± 3
MEL-HSA-P15%	15	182 ± 3	0.234 ± 0.03	262.4 ± 3	0.306 ± 0.03	−7.1 ± 0.4	81.19 ± 0.23	1.03 ± 0.01	5.60 ± 0.02	278 ± 2
MEL-HSA-P16%	16	231 ± 3	0.268 ± 0.02	304.3 ± 5	0.328 ± 0.04	−7.0 ± 0.2	79.46 ± 0.24	0.97 ± 0.01	5.52 ± 0.07	311 ± 4

**Table 3:** Obtained kinetic parameters of in situ thermogelling nasal formulations.

Kinetic Model	Kinetic Parameters	MEL-HSA-P12%	MEL-HSA-P13%	MEL-HSA-P14%	MEL-HSA-P15%	MEL-HSA-P16%
Zero order	k_0_ (μg min^−1^)	0.342	0.293	0.368	0.31	0.258
	R^2^	0.9265	0.9251	0.9904	0.9968	0.9976
	t_0.5_ (min)	342.31	292.61	357.98	310.02	257.76
First order	k_1_ × 10^−3^ (min^−1^)	1.487	1.126	1.254	1.092	0.974
	R^2^	0.9584	0.9586	0.9927	0.9723	0.9972
	t_0.5_ (min)	466.15	615.43	552.71	634.49	711.49
Higuchi model	k_H_ (μg min^−1/2^)	30.58	84.75	81.47	90.67	62.43
	R^2^	0.7954	0.7855	0.8708	0.8883	0.8684
	t_0.5_ (min)	427.58	718.23	663.67	822.44	389.75
Korshmeyer-Peppas model	k_K-P_ × 10^−2^ (min^−n^)	9.427	5.684	2.998	3.118	3.292
	n	1.05	1.11	1.23	1.21	1.17
	R^2^	0.6105	0.5516	0.7221	0.8396	0.7453
	t_0.5_ (min)	1043.99	1618.39	2047.18	1740.98	1848.54
Hixon-Crowell model	k_H-C_ (μg ^1/3^ min^−1^)	0.01	0.0046	0.0062	0.0043	0.0029
	R^2^	0.9838	0.987	0.9877	0.9863	0.9974
	t_0.5_ (min)	1610.35	2055.94	1950.68	2178.88	2466.87
Best fit		Hixon-Crowell model	Hixon-Crowell model	Zero order	Zero order	Zero order

## Data Availability

The data presented in this study are available on request from the corresponding author.

## Supplementary Materials

The following are available online at https://www.mdpi.com/article/10.3390/pharmaceutics13050646/s1, Figure S1: Primary data for the LCST and gelling time measurements, Figure S2. Drug release kinetics of in situ gelling formulations.
