# Supplementary material for: Development of In Situ Gelling Meloxicam-Human Serum Albumin Nanoparticle Formulation for Nose-to-Brain Application
Source: Pharmaceutics. 2021 May 1;13(5):646. doi: 10.3390/pharmaceutics13050646 (PMC8147280; doi:10.3390/pharmaceutics13050646)
Supplement: Supplementary file 1 [file pharmaceutics-13-00646-s001.zip › pharmaceutics-1177469-supplementary.pdf]

pharmaceutics

MDPI

Supplementary Materials: Development of In Situ Gelling Meloxicam-Human Serum Albumin Nanoparticle Formulation for Nose-to-Brain Application

Gábor Katona, Bence Sipos, Mária Budai-Szűcs, György Tibor Balogh, Szilvia Veszelka, Ilona Gróf, Mária A. Deli, Balázs Volk, Piroska Szabó-Révész and Ildikó Csóka

Citation: Katona, G.; Sipos, B.; Budai-Szűcs, M.; Balogh, G.T.; Veszelka, S.; Gróf, I.; Deli, M.A.; Volk, B.; Szabó-Révész, P.; Csóka, I. Development of In Situ Gelling Meloxicam-Human Serum Albumin Nanoparticle Formulation for Nose-to-Brain Application. *Pharmaceutics* **2021**, *13*, 646. https://doi.org/10.3390/pharmaceutics13050646

Academic Editor: Juan José Torrado

Received: 25 March 2021
Accepted: 28 April 2021
Published: 1 May 2021

Publisher's Note: MDPI stays neutral with regard to jurisdictional claims in published maps and institutional affiliations.

Copyright: © 2021 by the authors. Submitted for possible open access publication under the terms and conditions of the Creative Commons Attribution (CC BY) license (http://creativecommons.org/licenses/by/4.0/).

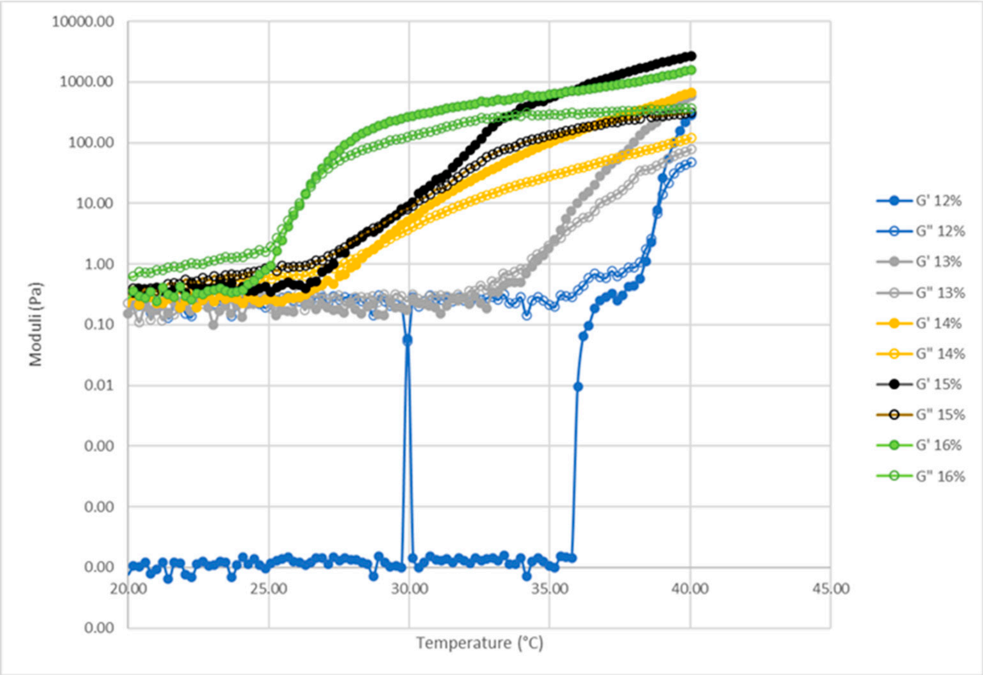

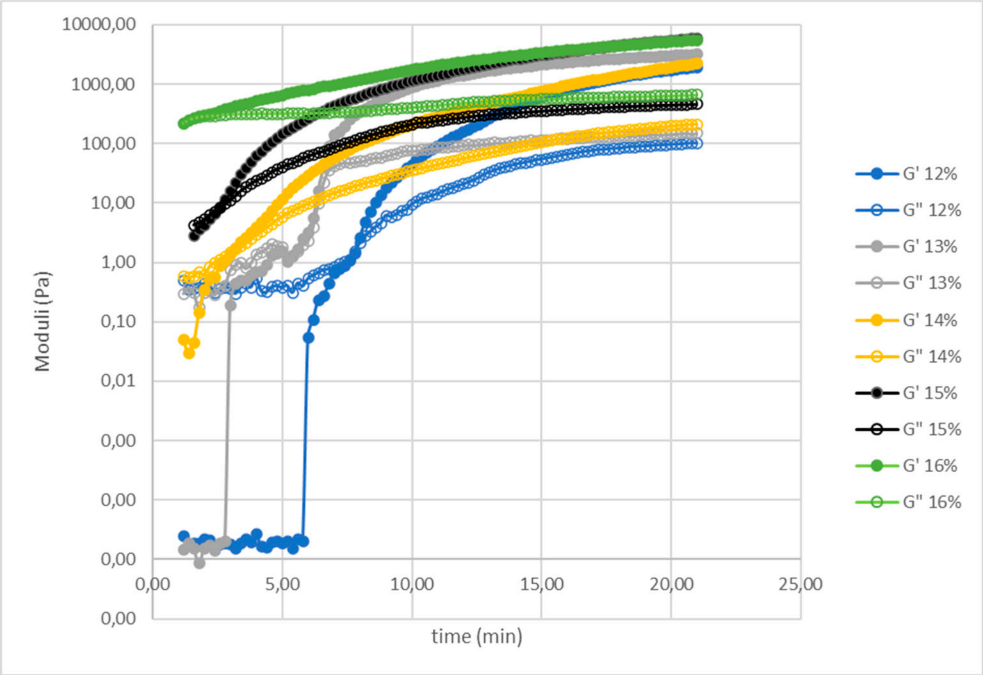

Figure S1. Primary data for the LCST and gelling time measurements.

MEL-HSA-P12%

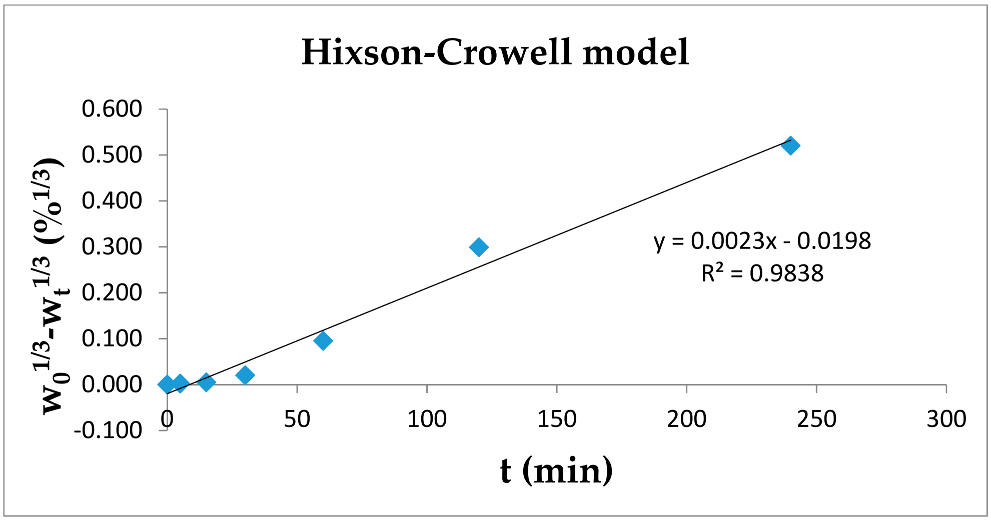

MEL-HSA-P13%

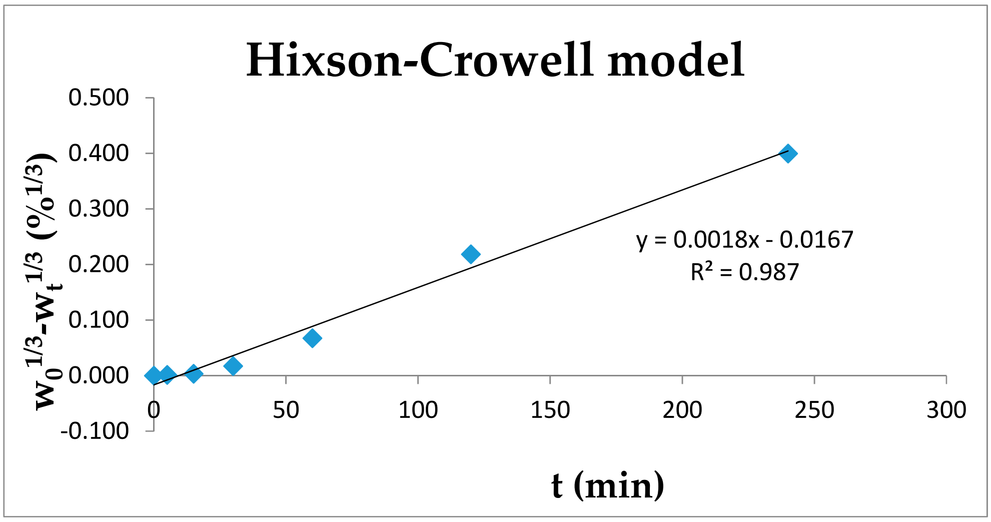

MEL-HSA-P14%

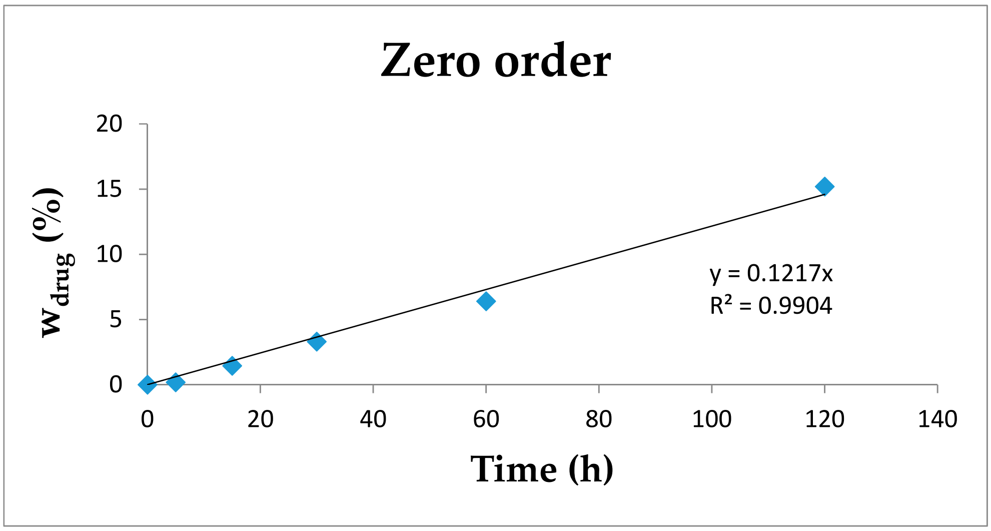

MEL-HSA-P15%

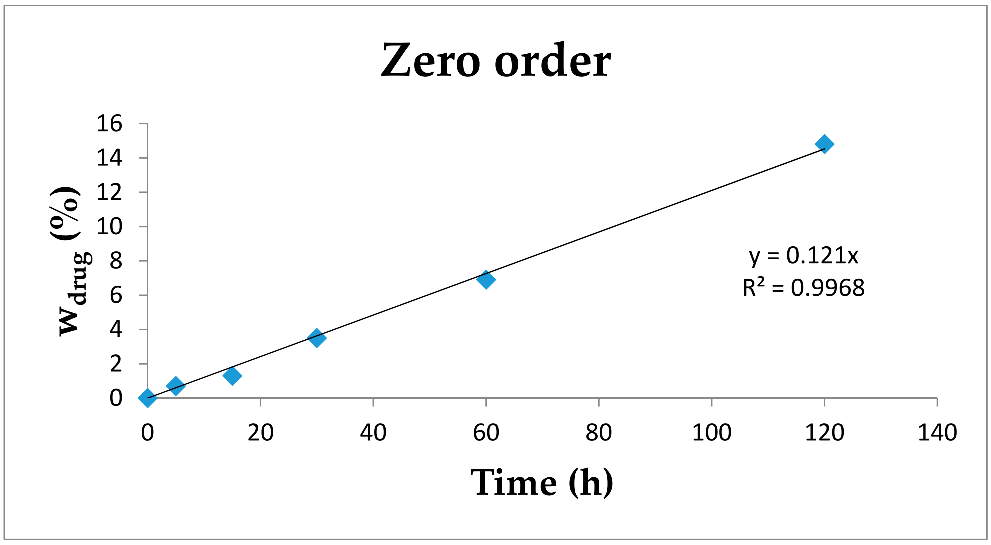

MEL-HSA-P16%

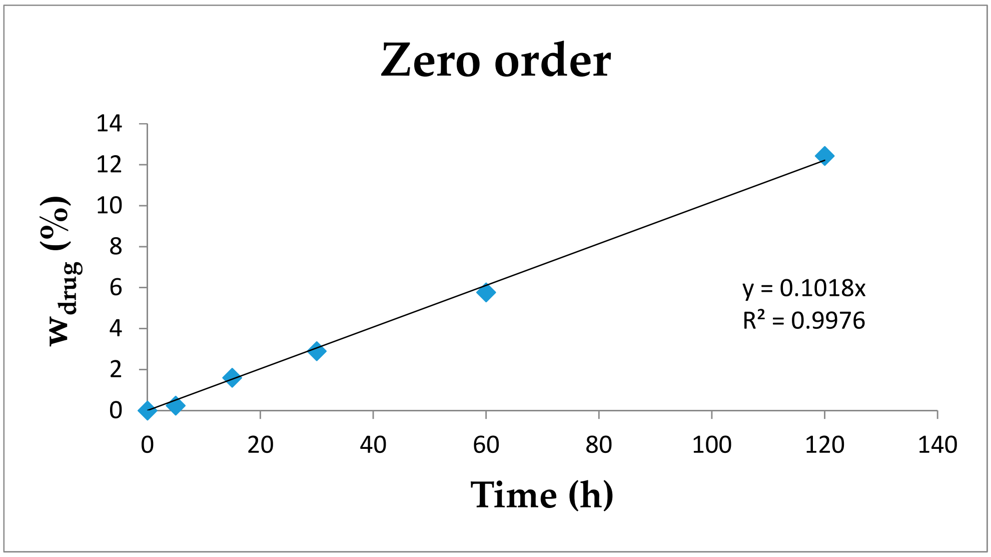

Figure S2. Drug release kinetics of in situ gelling formulations.
